# Recent Advances in Carbon-Based Materials for Adsorptive and Photocatalytic Antibiotic Removal

**DOI:** 10.3390/nano12224045

**Published:** 2022-11-17

**Authors:** Raner Ma, Yinghao Xue, Qian Ma, Yanyan Chen, Shiyin Yuan, Jianwei Fan

**Affiliations:** State Key Laboratory of Pollution Control and Resources Reuse, College of Environmental Science and Engineering, Tongji University, Shanghai 200092, China

**Keywords:** carbon-based materials, antibiotics, adsorption, photocatalysis, graphene, carbon nanotube, biochar, hierarchical porous carbon

## Abstract

Antibiotics have been a primary environmental concern due to their widespread dispersion, harmful bioaccumulation, and resistance to mineralization. Unfortunately, typical processes in wastewater treatment plants are insufficient for complete antibiotic removal, and their derivatives in effluent can pose a threat to human health and aquatic communities. Adsorption and photocatalysis are proven to be the most commonly used and promising tertiary treatment methods. Carbon-based materials, especially those based on graphene, carbon nanotube, biochar, and hierarchical porous carbon, have attracted much attention in antibiotic removal as green adsorbents and photocatalysts because of their availability, unique pore structures, and superior physicochemical properties. This review provides an overview of the characteristics of the four most commonly used carbonaceous materials and their applications in antibiotic removal via adsorption and photodegradation, and the preparation of carbonaceous materials and remediation properties regarding target contaminants are clarified. Meanwhile, the fundamental adsorption and photodegradation mechanisms and influencing factors are summarized. Finally, existing problems and future research needs are put forward. This work is expected to inspire subsequent research in carbon-based adsorbent and photocatalyst design, particularly for antibiotics removal.

## 1. Introduction

There have been growing concerns over water pollution due to human activities and technological and industrial development. Because some synthetic organic chemicals pose a significant risk to the ecological environment or human health while being used extensively, they have attracted the attention of researchers, and are defined as emerging contaminants (ECs) [1,2]. ECs encompass a wide range of artificial chemicals, such as pharmaceutical and personal care products (PPCPs), endocrine-disrupting compounds (EDCs), surfactants, and pesticides, among others [3,4,5,6]. They are difficult to remediate by natural attenuation and conventional treatment processes, so they easily accumulate in the ecosystem [7].

The excess presence of pharmaceuticals in water is emerging contamination, and antibiotics are one of the most common treatment groups. Antibiotics refer to metabolites produced by microorganisms such as fungi, bacteria, and actinomycetes that impede the growth and reproduction of other microbes [8,9]. Antibiotics are extensively used in aquaculture and human/animal therapies, resulting in a direct discharge of antibiotic residue into water sources, causing pollution problems [10]. Due to the antibacterial ability arising from functional groups of antibiotics, such as the linear fused tetracyclic nucleus (four rings) of tetracyclines, four-membered β-lactam ring of β-lactams, and large lactone ring of macrolides, the degradation of antibiotic molecules by microorganisms is beset with difficulties [11]. It was reported that antibiotics existing in sediments, soil, and water not only bring biochemical pollution, but also hasten the formation of antibiotic-resistance genes (ARG) and antibiotic-resistant bacteria (ARB) [12]. Therefore, the removals of antibiotics and their derivatives from water environments are urgent. Research showed that sulfonamides are the most frequently detected in all kinds of water environments, followed by fluoroquinolones, macrolides, and tetracyclines, while other categories of antibiotics are not as frequently detected [13]. Therefore, the removal of these four groups of antibiotics has attracted most researchers’ attention.

Various techniques have been devised to remove or reduce the concentration of antibiotics in the water environment, ranging from advanced oxidation such as ozonation, Fenton and photo-Fenton processes, and electrochemical oxidation to biological, photocatalysis, chlorination, membrane separation and adsorption [11,14,15,16]. Among them, adsorption is especially attractive for its facile operation, high efficiency, and potential scaling up [17]. As a green AOP technology, photocatalysis is expected to use the strong oxidation ability of photogenerated holes to mineralize antibiotic pollutants completely, without forming intermediates [18,19].

Both the efficiency of the adsorption and the photocatalysis process is strongly influenced by the type of materials and their characteristics. Compared to other commonly used adsorbents and photocatalysts, carbon-based materials show more advanced properties, including large specific surface areas (SSAs), developed pore structures, abundant functional groups, and high chemical stability [20]. Until now, many efforts have been made in pore structure engineering that can be classified as hierarchical porous structures, interconnected pores, carbonaceous defects, and graphene assemblies. In addition, more researchers are focusing on doping carbonaceous materials with heterogeneous atoms such as B, N, S, O, and P, and loading functional compounds like metals (oxides) to enhance the performance of adsorbents/catalysts for targeted pollutant removal [21,22,23]. As a result, carbon-based materials have been widely applied in both treatment processes in the past few years, showing great potential in applications of antibiotic removal. 

**Figure 1:**
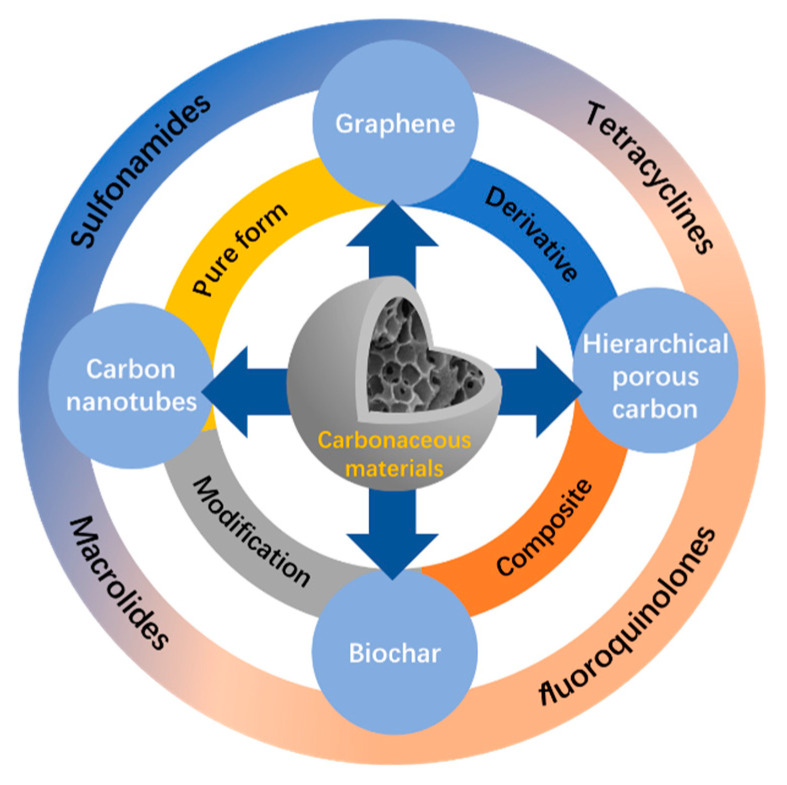
Antibiotics removal on carbon-based materials.

In this review, we focus on removing antibiotics via adsorption and photodegradation by typical carbon-based materials. We consider graphene, carbon nanotube, biochar, and hierarchical porous carbon as the four primary carbonaceous materials, discussing their preparation and remediation properties regarding each target contaminant (Figure 1). Meanwhile, the basic adsorption and photodegradation mechanisms and influencing factors are summarized. Finally, existing problems and future research needs are put forward. We expect this review to inspire subsequent research in carbon-based adsorbent and photocatalyst design, particularly for antibiotics removal.

## 2. Characteristics of Carbonaceous Materials

The main carbonaceous materials used for antibiotic removal are graphene, carbon nanotube (CNT), biochar (BC), and hierarchical porous carbon (HPC), which is a novel, precisely designed carbonaceous material. They have many advantages, such as simple synthesis methods, cheap and readily available raw materials, large porosity, high SSA, well-developed and adjustable pore structure consisting of micropores, mesoporous, and macropores, and a large number of oxygen-containing functional groups, so they have many potential application prospects in antibiotic removal.

### 2.1. Graphene and Its Derivatives

Graphene is a two-dimensional nanomaterial in which carbon atoms connected by sp^2^ hybridization are closely packed into a single atomic layer of honeycomb-like crystal lattice which is organized through σ- and π-bonds. This material is the basis for all forms of graphite: folded-up graphene becomes fullerene (0D), a rolled-up graphene layer forms a carbon nanotube (1D), and graphite (3D) sheets become graphene when taken apart. It has the characteristics of good stability, conductivity, optical properties, and adsorption capacity, which makes graphene-based materials a new type of environmentally friendly catalysts with more active sites. 

**Figure 2:**
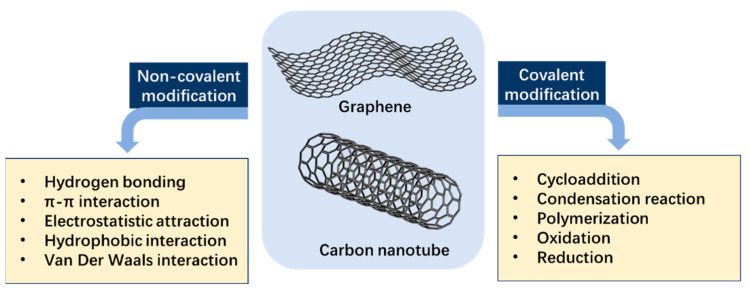
Schematic representation of modifications of graphene and carbon nanotube by covalent and non-covalent methodology.

Synthesis approaches have a crucial impact on the performance and quality of graphene, and there are different synthetic routes such as chemical vapor deposition, the exfoliation method, and the organic synthesis approach [24]. These synthesis methods can be divided into two approaches: the top-down approach, based on reducing van der Waals forces of attraction between the graphite layers, and the bottom-up approach, based on combining unique molecular building blocks. In its pure form, graphene cannot perform well in water due to its hydrophobic nature. Thus, materials with hydrophilic properties, such as graphene oxide (GO) and reduced graphene oxide (rGO), are prepared for water treatment purposes. GO is graphene modified with oxygen-containing functional groups, showing various active sites (hydroxyl, carboxyl, epoxy, and carbonyl) in the carbon lattice. rGO is a type of GO with lesser oxygen content and higher interaction with the π bond of organic molecules, making it more efficient to remove the contaminants in the water. The graphene can be functionalized through covalent and noncovalent bonding to augment its loading capacity, specificity, biocompatibility and solubility (Figure 2), and graphene-based composites were invented to improve its binding affinity for anionic compounds and ability to be detached from treated wastewater.

Graphene-based materials are widely used in wastewater treatment via separation, membrane, and degradation processes. They are proven to be incredibly effective for the adsorption of inorganic contaminants such as heavy metal ions and rare earth metal ions, as well as organic contaminants, especially dyes, antibiotics [25], and AOPs such as the Fenton process, electro-Fenton process, photocatalysis, photoelectrocatalysis, ozonation, and sonolysis [26]. 

### 2.2. Carbon Nanotube (CNT)

A carbon nanotube, several microns in length, is a hollow cylindrical tube with side walls composed of sp^2^ hybridized carbon atoms, including two types: single-walled carbon nanotubes (SWCNTs) and multi-walled carbon nanotubes (MWCNTs). SWCNTs with a diameter between 0.4 and 3 nm are formed by curling a single layer of graphene. MWCNTs, whose outer and inner diameters are 2–100 nm and 1–3 nm, respectively, are made of two or more graphene sheets rolling into cylindrical shapes [27]. Due to of the attractive properties of CNTs, such as large surface area-to-volume ratio, high thermal and chemical stability, well-defined adsorption sites, easy attachment of functional groups, and nanoparticle loading [28], much research has been conducted to develop its potential in wastewater treatment applications.

CNTs are produced by using carbon sources and energy. Generally, there are three main synthesis methods: (1) the arc discharge method between graphite electrodes, (2) the laser ablation method catalyzed by transition metals, and (3) the chemical vapor deposition method for bulk fabrication [27]. Due to the defects of traditional CNTs, such as easy entanglement and agglomeration, surface inertness, and dispersing difficulty in the polymer matrix, it is hard to exert their decontamination performances, so their application is greatly restricted [29]. Therefore, similarly to graphene, functional methods based on covalent and noncovalent bonding are applied to equip CNTs with the required functional groups and make them more dispersible, increasing their affinity for water contaminants (Figure 2). Surface-modified CNTs have been studied extensively to remove heavy metals and organics from wastewater by adsorption. Furthermore, CNT composites can be prepared by introducing nanoparticles, high molecular polymers, etc., as photocatalysts and electrocatalysts (electrochemical reaction electrodes, microbial dye battery electrodes).

### 2.3. Biochar (BC)

Biochar is the solid carbonaceous residue from various agricultural and industrial biomasses via thermal methods. It is a low-cost, porous, and stable carbonaceous material with high SSA, high surface activity, and high ion exchange capacity that is well suited for use as an adsorbent and as a support matrix for metals and metal oxides [30,31].

As a biomass-derived material, BC is fabricated from different biomass feedstocks, including agricultural residues, forestry waste, organic fraction of municipal solid waste, industrial biomass by-products, and animal manures, via pyrolysis (300–700 °C) under oxygen-free or oxygen-limited conditions [32]. Critical physical properties of BC, surface area, and porosity play crucial roles in the wastewater treatment process. Moreover, biomass characteristics, including morphology structure, particle size, and pyrolysis parameters, predominantly temperature, are key influencing factors [33]. Higher pyrolysis temperature (>500 °C) results in lower oxygen content, greater hydrophobicity, higher surface area, and higher micropore volume, making the produced BC more suitable for removing organic pollutants. Conversely, BC pyrolyzed under lower temperatures (<500 °C) is better fit for the removal of inorganic pollutants [34]. 

To enable BC to better fulfill the requirements of practical applications in pollutant removal, modification is widely used to improve its adsorption or catalysis performance and alleviate secondary pollution. Physical modification methods, including steam/gas activation (air, CO_2_), ball milling, and microwave pyrolysis, have been applied to develop the pore structure of BC, and enhance its hydrophobicity and stability [35]. Chemical modification methods involving the action of chemical compounds can increase BC’s surface functional groups, polarity, and porosity. Acids, H_2_O_2_, and KMnO_4_ are common oxidants, while alkalis are reducers [35]. BC-based composites prepared by combining BC with clay minerals, metals, metal oxides, carbon materials, and polymers via impregnation, co-pyrolysis, or co-precipitation offer enhanced adsorption capabilities and conduct particular functionalities in contaminants removal [36]. In general, loaded magnetic materials enable BC to have magnetic separation properties, loaded carbonaceous materials improve their porosity, loaded TiO_2_ makes it catalytic, and loaded nano zero-valent iron can make it highly reactive.

### 2.4. Hierarchical Porous Carbon (HPC)

Hierarchical porous carbon is a novel carbonaceous material that contains two, or all three, types of interconnected pores (micropores (<2 nm), mesopores (2–50 nm), and macropores (>50 nm)), which serve different functions. Micro and smaller mesopores can provide a large surface area to promote active site dispersion, larger mesopores can minimize diffusive resistance to mass transport and allow easy access to the micropores, and macropores shorten mass-transfer pathways as reactant reservoirs. Moreover, the interconnected hierarchical pore structures can facilitate reactant entry into micropores and expedite the reaction rate [37,38]. In conclusion, because the space in HPC is effectively utilized, HPC with multiple levels of pores has higher SSA and pore volume than carbon materials with single-type pores [39,40]. It exhibits excellent performance in applications like adsorption, catalysis, and energy storage due to the functional synergy of the above structures.

The typical preparation strategies of HPC are the hard/soft template method and the template free method [41]. The hard template method synthesizes HPC materials by using silica, metal oxides, inorganic salts, and sublimable organic compounds as hard templates, incorporating them into carbons or carbon precursors, and removing them by either chemical etching or dissolution. Unlike hard templates, soft templates are mainly organic molecules or block copolymers that can be deposited or evaporated during annealing, avoiding the template removal step after pyrolysis. The template free method fabricates HPC by pyrolyzing select carbon precursors, mainly biomass and pre-engineered organic matter with intrinsic macropores, and constructing hierarchical porous structures by chemical activation. There are also some modification methods for HPC to develop additional micropores and mesopores, such as a combination of template carbonization and chemical activation (i.e., KOH or NaOH) to optimize the synthesis process and treat precursors with different chemicals [39]. Changing the feedstock to heteroatom containing materials to prepare heteroatom-doped HPC can change its physicochemical and electronic properties, and enhance its adsorption, catalysis, and electrochemistry performance [37].

Some researchers have paid attention to the application of HPC in removing organic contaminants from wastewater by adsorption. They studied organic contaminants included dye [42,43], antibiotic [44,45], hydrocarbon [46,47], bilirubin [48], etc.

## 3. Adsorption Removal of Antibiotics by Carbon-Based Materials

There has been extensive research on using carbonaceous materials to remove antibiotics by adsorption. Adsorption is an exothermic process that occurs on a surface, and involves the accumulation of adsorbate molecules in a gaseous or liquid form. Adsorption on carbonaceous materials includes four steps: (1) transport of solute from the bulk, (2) diffusion through the liquid film around the porous carbon particle, (3) diffusion in the liquid contained in the pore space, and (4) interaction between adsorbate and adsorbent [49]. The adsorption interactions between carbon-based materials and antibiotics can be summarized into physical and chemical adsorption. Weak Van der Waal interactions, π-π interactions, electrostatic contacts, and pore-filling/size-selective adsorption are the main mechanisms of physical adsorption. For chemical adsorption, surface chemical bonds or inner sphere coordination complexes are formed on an adsorbate and adsorbent surface either by electron pairing or electron transfer via Lewis acid-base interaction, ion/ligand exchange, and oxidation or reduction [50]. The existence of functional groups (–COOH, –OH, –NH_2_) in the carbonaceous materials may also have an impact on the adsorption process and efficiency [51]. The adsorption of antibiotics from the aqueous phase by carbonaceous materials is mainly influenced by three factors: the functional groups in the carbon materials, the pore size and pore uniformity of carbon materials, and environmental parameters, including pH, temperature, the concentration of inorganic salt, and other organic substances in the aqueous solution [52].

### 3.1. Graphene-Based Materials Applied in Antibiotics Adsorption

Several excellent properties make graphene an effective adsorbent for removing antibiotics.: (1) highly exposed atoms lead to easy contact with antibiotics (mainly by π-π interaction), (2) porous structure and high surface area ensure faster diffusion or surface reactions of antibiotics, resulting in rapid and effective adsorption, and (3) lower cost of large-scale production [53]. The adsorption of GO, rGO, functionalized graphene, and graphene-based composites on antibiotics will be presented and discussed below. Table 1 lists recent studies of graphene-based adsorbents on antibiotics.

GO has been investigated extensively for antibiotic adsorption removal, particularly in aqueous environments due to its high hydrophilicity. It has been reported that GO can effectively sorb sulfamethoxazole (SMX), ciprofloxacin (CIP) [54], and various tetracycline antibiotics [55] via π-π interaction and electrostatic attractions. Recently, Salihi et al. (2020) reported on the adsorption behaviors of trimethoprim (TMP) and isoniazid (INH) onto GO. The maximum adsorption capacities of TMP and INH were 204.08 mg g^−1^ (pH = 8) and 13.89 mg g^−1^ (pH = 2), respectively [56]. Moreira et al. (2020) verified the simultaneous adsorption degradation of norfloxacin (NOR) by GO, with a removal capacity of 374.9 mg g^−1^ [57]. Moreover, GO also exhibited good adsorption properties for cephalexin (CFX) (164.35 mg g^−1^)[58] and azithromycin (AZM) (55.55 mg g^−1^) [59]. Fewer studies were conducted on rGO. For example, Liu et al. (2016) examined the adsorption of two sulfonamide antibiotics by two rGOs, which are less affected by dissolved organic matter than CNTs and graphite [60].

For better adsorption properties, hydrophilic functional groups (–OH, epoxide, –COOH) are often used for the covalent modification of graphene, and graphene-based composites were prepared by heteroatom doping [61], nanoparticle loading, and organic molecular and polymeric modification [62]. Recently, different types of components are often used simultaneously in modifications to achieve a synergistic effect, among which magnetic nanoparticles such as Fe_3_O_4_ are most commonly used to obtain good solid-liquid separation properties [62,63]. For instance, Lin et al. (2021) fabricated an ethanolamine and Fe_3_O_4_ nanoparticle; a co-functionalized, graphene-based adsorbent with magnetic properties by co-precipitation. The introduction of ethanolamine into GO can decrease the possibility of agglomeration, while Fe_3_O_4_ nanoparticles can make the adsorbent rapidly separate using a magnetic field. Due to the synergy, in only five minutes, the adsorption capacities of the material for TC and levofloxacin (LVX) were 315.25 mg g^−1^ and 229.53 mg g^−1^ [64]. In addition, natural materials like chitosan [65] and attapulgite [66] have also been used to modify graphene to adsorb antibiotics. 

To overcome the shortcomings of the 2D nanostructure, three-dimensional (3D) graphene materials were synthesized, including aerogels and hydrogels. A ring defects-rich and pyridinic N-doped graphene aerogel was fabricated by Wang et al. (2022), and exhibited an outstanding performance for TC removal (adsorption capacity of 607.1 mg g^−1^) as a floating adsorbent [67]. Moreover, ultra-thin g-C_3_N_4_ modified graphene oxide hydrogels were prepared via a one-step hydrothermal reaction by Wang et al. (2022), which exhibited super high co-adsorption capabilities for TC and Cu(II) [68]. These aerogels and hydrogels have large adsorption capacity and can be easily modified and separated from water.

MOFs/GO composites are also becoming promising adsorbents for wastewater treatment, as the metal ions in MOFs can interact with the epoxy and hydroxyl functional groups on both sides of the GO flakes. Chen et al. (2022) synthesized Alg-Cu@GO@MOF-525 by dispersing GO with copper alginate and in situ growth of MOF-525, which helped reduce aggregation. The SSA of the material was as high as 807.3 m^2^ g^−1^, which is favorable for the adsorption of TC (adsorption capacity of 533 mg g^−1^) [69].

**Table 1:** Adsorption characteristics of antibiotics by graphene-based materials.

Antibiotic Class	Antibiotic Compounds	Adsorbent	Temperature (K)	pH	Equilibrium Time (Min)	Adsorption Capacity Q_m_ (mg g^−1^)	Refs.
Tetracycline	Tetracycline	GO	298	3.6	190	313.48	[55]
	Tetracycline	MAEGO	303	4	1440	487.82	[64]
	Tetracycline	GO@ATP	308	5	120		[66]
	Tetracycline	DNGA	298	4	20	607.1	[67]
	Tetracycline	UCN-GH	298	5	100		[68]
	Tetracycline	Alg-Cu@GO@MOF-525	318	7	900	533.2	[69]
	Oxytetracycline	GO	298	3.6	90	212.31	[55]
	Oxytetracycline	GO	293	5	60	130.4	[61]
	Oxytetracycline	B-rGO	293	5	60	83.9	[61]
	Doxycycline	GO	298	3.6	90	398.41	[55]
	Doxycycline	GO@Fe_3_O_4_@β-cyclodextrin	298	7	45	204.5	[70]
Sulfonamide	Sulfamethoxazole	GO	298	5	1440	240	[54]
	Sulfamethoxazole	GO@β-cyclodextrin@ dopamine hydrochloride	308	2	90	144	[62]
	Sulfadiazine	GO@β-cyclodextrin@ dopamine hydrochloride	308	2	90	152	[62]
	Trimethoprim	GO	298	8	60	204.08	[56]
	Isoniazid	GO	298	2	60	13.89	[56]
Quinolone	Ciprofloxacin	GO	298	5	2880	379	[54]
	Norfloxacin	GO	303	7	30	374.9	[57]
	levofloxacin	MAEGO	303	4	1440	330.71	[64]
β-lactams	Cephalexin	GO	298	7	420	164.35	[58]
Macrolide	Azithromycin	GO	298	7	0.25	55.55	[59]

### 3.2. Carbon Nanotube-Based Materials Applied in Antibiotics Adsorption

The large SSA, biological harmlessness, and an abundance of oxygen-containing functional groups of CNTs offered various potential applications for the adsorption of antibiotics in an aqueous environment. Table 2 illustrates data on antibiotic removal by various CNTs.

Earlier research focused on using pristine and oxidized MWCNTs to adsorb different kinds of antibiotics, such as sulfonamides [71], amoxicillin [72], fluoroquinolone [73], and ciprofloxacin [74], and comparing the adsorption effect of different types of CNTs towards antibiotics. In most instances, SWCNT showed the highest adsorption capacity for its large surface area and unique basic surface [74,75]. 

CNT-based composites have been studied extensively for the adsorption removal of antibiotics, especially for TC and CIP. Various magnetic carbon nanotube adsorbents were developed via coprecipitation [76,77,78] or hydrothermal [79] methods for their magnetic separation ability, which could improve the recyclability of CNTs. For example, Sereshti et al. (2022) developed Fe_3_O_4_ doped and CdS functionalized MWCNTs, used for TC and cefixime removal. Due to the introduction of Fe_3_O_4_, the material was magnetized and could be easily separated by an external magnet [78]. However, adsorption site occupation by ferrites may lead to a decrease in adsorption capacity. Zhao et al. (2021) used three high crystalline magnetic nano spinel ferrites (MFe_2_O_4_; M = Co, Cu, Mn) to modify carbon nanotubes and compared their adsorption mechanisms. They found that the highest adsorption capacity (63.32 mg g^−1^) and removal efficiency (99.3%) of CIP were obtained by using CoFe_2_O_4_/CNTs, because Co atoms were most favorable for O adsorption according to their d-band states [79]. Besides, layered double hydroxides (LDHs) [80] and ionic liquids (ILs) proved to be ideal materials to be loaded into CNTs for better removal efficiency.

Fe-doped MOF-loaded CNTs [81,82] attracted many researchers’ attention, as the introduction of high-valence Fe^3+^ metal ions could improve the water stability of MOFs, therefore enhancing their applications in the liquid phase. The CNT/GO/sodium alginate triple-network nanocomposite hydrogel was a 3D material that was prepared by Ma et al. (2020). It achieved high-efficiency removal of CIP, with an adsorption capacity of 200 mg g^−1^ at 288 K [83].

**Table 2:** Adsorption characteristics of antibiotics by CNT-based materials.

Antibiotic Class	Antibiotic Compounds	Adsorbent	Temperature (K)	pH	Equilibrium Time (Min)	Adsorption Capacity Q_m_ (mg g^−1^)	Refs.
Tetracycline	Tetracycline	M-MWCNT	308	4-7	240	494.91	[76]
	Tetracycline	Fe_3_O_4_/MWCNT-CdS	298	5	60	116.27	[78]
	Tetracycline	MWCNT/ZIF-8(Fe)	298	6	360	589.42	[82]
	Tetracycline hydrochloride	LDH@CNT	298	8	480	756.2	[80]
	Tetracycline hydrochloride	MWCNT/MIL-53(Fe)	298	7		364.37	[81]
	Oxytetracycline hydrochloride	MWCNT/MIL-53(Fe)	298	7		325.59	[81]
	Chlortetracycline hydrochloride	MWCNT/MIL-53(Fe)	298	7		180.68	[81]
Sulfonamide	Sulfamethoxazole	MWCNT	298	3	720		[71]
	Sulfamethazine	Fe_3_O_4_/MWCNTs	298	7	1440		[77]
Quinolone	Fluoroquinolone	O-MWCNT	298	3	1440		[73]
	Ciprofloxacin	CoFe_2_O_4_/CNTs	298	6–7	300	63.32	[79]
	Ciprofloxacin	CNTs/L-cys@GO/SA	288	5.4	3600	200	[83]
	Ciprofloxacin	4.7%O-MWCNT	298	4	60	177.8	[84]
β-lactams	Amoxicillin	MWCNT	333	7	75	159.4	[72]
Nitroimidazole	Metronidazole	SWCNT	298	7	7200	101	[75]
	Dimetridazole	SWCNT	298	7	7200	84	[75]
Cephalosporin	Cefixime	Fe_3_O_4_/MWCNT-CdS	298	5	60	105.26	[78]

### 3.3. Biochar-Based Materials Applied in Antibiotics Adsorption

Due to its excellent properties, e.g., high porosity, aromaticity, multiple anionic functional groups, large surface area, and a wide range of sources, biochar has been considered an effective carbonaceous sorbent for antibiotics. Table 3 lists recent advances in BC-based materials for the sorptive removal of antibiotics.

The adsorption of TC on BC derived from auricula dregs [85] and cow manure [86], obtained at different pyrolysis temperatures, was investigated. Results showed that the BCs prepared at a high temperature had a greater capacity for removing TC, as they have better pore distribution, larger SSA, and greater adsorption affinity. Stylianou et al. (2021) assessed the adsorption capacity of three different derived biochar on seven antibiotics. They found that the removal efficiency of these antibiotics dramatically depends on the pH value of the medium and, therefore, on the antibiotics’ speciation, and the physicochemical and structural characteristics of the adsorbents (primarily SSA, pore size, functional groups, dosage) [87]. Zhao et al. (2022) used a concentrated sulfuric acid-based, one-step carbonization method to synthesize BC for enrofloxacin (ENR) adsorption. The obtained BC was highly graphitized with more active functional groups on the surface and showed significantly superior adsorption capacity for ENR (142.3 mg g^−1^), which was 13.7 times that of pyrolytically synthesized BC (10.4 mg g^−1^) [88]. In conclusion, antibiotics adsorption by pristine BCs mainly depends on their feedstock type, synthesis method, and synthesis condition. 

Various modification methods have been used to improve the unsatisfactory adsorption capacities of pristine BCs. Ball milling, a physical method, has received enormous attention from researchers recently. For example, urea N-doped biochar was facilely prepared by Wu et al. (2021) via wet ball-milling to adsorb and degrade NOR. It was proved that ball-milling enhanced biochar for NOR adsorption (adsorption capacity of 11.48 mg g^−1^) by H-bonds, π-π EDA, and pore-filling [89]. At the same time, different kinds of chemical reagents were used to change the chemical nature of BCs, such as H_3_PO_4_ [90,91], citric acid [92], and ZnCl_2_ [93,94], all of which confirmed that chemical modification could increase the functional groups, SSA, and porosity of BC. Specifically, Sheng et al. (2022) synthesized a critical acid-modified BC (CABRC) as an efficient adsorbent for TC. Higher surface area, more pore structures, and stronger electron donor capacity provided by generated oxygen functional groups (e.g., –COO, –COOH, and –OH) synergistically improved the adsorption performance of the CABRC [92]. 

FeCl_3_ was widely used to synthesize magnetic BCs via the impregnation method, and some special chemical reagents were added for better adsorption capacities and higher chemical stability, such as humic acid for coating [95], dicyandiamide for the introduction of N [96], MgCl_2_·6H_2_O [97], and NiSO_4_·6H_2_O [98] for the formation of ferrites. Apart from FeCl_3_, K_2_FeO_4_ was also found suitable for BC magnetization, as ferrates could not only serve as the precursor of iron oxide (loaded on the biochar surface) but also enlarge the surface area of the resulting biochar due to their solid oxidizing capability [99,100]. In addition, Qu et al. (2021) studied a novel magnetization process. They used two-step Microwave (MW) assisted processes to provide the obtained BC with high SSA and favorable magnetism, whose adsorption capacity of TC was 202.62 mg g^−1^ [101]. 

Furthermore, clay mineral BC composites are known to have improved overall performance [102,103]. For instance, Arif et al. (2022) investigated the removal of CIP using biochar treated with clay minerals and subsequently activated with carbon dioxide. The material exhibited a large adsorption capacity (50.32 mg g^−1^) due to enhanced active sites and functional groups [103]. In another research, Xiang et al. (2022) improved the SSA and pore volume of two biochar by lignin impregnation and enhanced their adsorption capacities of tetracycline hydrochloride (TCH). The results illustrated that biochar with porous modulation by lignin impregnation could be significantly applied to the removal of antibiotics [104].

**Table 3:** Adsorption characteristics of antibiotics by BC-based materials.

Biomass	Engineering Method	Antibiotic	Pyrolysis Temp (°C)	Adsorption Temp (K)	pH	Equilibrium Time (Min)	Adsorption Capacity Q_m_ (mg g^−1^)	Refs.
Auricula dregs		Tetracycline	700	298	7	60	11.9	[85]
Cow manure		Tetracycline	700	298	6	1440	11.80	[86]
Camellia oleifera shells	H_3_PO_4_	Tetracycline	600	298	6	240	451.6	[91]
Biogas residue	Citric acid	Tetracycline	800	298	7	600	58.25	[92]
Aerobic granular sludge	ZnCl_2_	Tetracycline	700	308	5	2880	93.44	[93]
Flueggea suffruticosa	ZnCl_2_	Tetracycline	500	303	7	50	188.7	[94]
Walnut shell	FeCl_3_·6H_2_O, dicyandiamide	Tetracycline	600	298	7.2		238.9	[96]
Water hyacinth	FeCl_3_·6H_2_O	Tetracycline	700	318		200	202.62	[101]
Wheat Straw	Lignin	Tetracycline hydrochloride	600	298	7		31.48	[104]
Flueggea suffruticosa	ZnCl_2_	Chlortetracycline	500	303	10		200.0	[94]
Flueggea suffruticosa	ZnCl_2_	Oxytetracycline	500	303	7		129.9	[94]
Coffee grounds	H_3_PO_4_	Sulfadiazine	700	298		180	139.2	[90]
Wheat stalk	K_2_FeO_4_	Sulfadiazine	700	298	6	540	47.85	[100]
Garlic peel	Concentrated H_2_SO_4_ carbonization	Enrofloxacin		298	7	T_1/2_ = 34.13	142.3	[88]
Apple branches	FeCl_3_, humic acid	Enrofloxacin	700	308	5	720	48.3	[95]
Apple branches	FeCl_3_, humic acid	Moxifloxacin	700	308	8	720	61.5	[95]
Corn stalk	Ball-milling, urea	Norfloxacin	600	298	5		11.48	[89]
Sludge	Bentonite	Norfloxacin	550	298	6	1080	89.36	[102]
Pomelo peel	MgFe_2_O_4_	Levofloxacin	700	298	5	240	115	[97]
Vinasse	NiFe_2_O_4_	Levofloxacin	700	298	6	1080	172	[98]
Rice husk	Montmorillonite, CO_2_	Ciprofloxacin	350	295	7	720	50.32	[103]
Penicillin fermentation dregs	Acetic acid, K_2_FeO_4_	Penicillin	400	308	11		322.58	[99]

### 3.4. Hierarchical Porous Carbon-Based Materials Applied in Antibiotics Adsorption

Recently, HPC with uniform and interconnected pore structure for antibiotics adsorption has received increasing research attention (Table 4), which is mostly due to the special characteristics of HPC, including its fine and well-defined pore systems, good mass transport through larger pores, and the substantial number of adsorption sites at smaller pores [39].

Most HPC for antibiotics adsorption were synthesized via the template free method, using a two-step thermal treatment involving low-temperature carbonization, which uses biomass as carbon precursor, and high-temperature activation, which includes acid-alkaline treatment, metal ions, steam/gas purging, or oxidizing agents [44,105,106,107]. Wang et al. (2022) chose five waste biomasses to prepare hierarchically micro/mesoporous carbons with a high BET surface area of 1687-2003 m^2^ g^−1^ and large adsorption capacity for chloramphenicol (CAP) (>300 mg g^−1^) after an equilibrium of only 40 min. They established that micropore filling might be a leading adsorption mechanism and that the ratio of micropores to mesopores in biochar adsorbents was a key element in attaining rapid, high-capacity adsorption [44]. To simplify the manufacturing process and reduce preparation cost and time, Chen et al. (2021) proposed a one-step method for making layered carbons with different pore structures by adjusting the temperature and the mass ratio of activator K_2_CO_3_. The decomposed K_2_O and CO_2_ reacted with carbon, forming more micropores and improving pore volume. The obtained HPC had excellent adsorption capacities (534 mg g^−1^) at a lower initial concentration of CAP (120 mg L^−1^) [108]. Other researchers used pre-engineered organic matter as a carbon precursor. Pi et al. (2022) used glucose hydrochar as the precursor to prepare bifunctional N-doped HPC, which could adsorb TC and activate PDS as the activator, maintaining above 77.18% adsorption capacity after six cycles. The material’s large BET surface area and microporous-mesoporous structure promoted adsorption by offering more binding sites on its surface to form a stable interaction. At the same time, the mesoporous was conducive to the transfer of reactants or electrons and increased the catalytic performance of the material [45].

Earlier researchers used halloysite nanotubes as the hard template to obtain HPC through liquid phase impregnation and carbonization. This kind of HPC possessed extraordinary adsorption capacity for TC (1297.0 mg g^−1^) and CAP (1067.2 mg g^−1^) [109,110]. In another study, a soft-hard template method was used to fabricate an HPC that possessed micro/meso bimodal pores, in which tetraethyl orthosilicate was used as an inorganic precursor, and the triblock copolymer F127 as a structure-directing agent. Due to the bimodal porous structure with meso channels and connected micropores of the prepared HPC, which enabled rapid transport of TC molecules and easier access to the adsorption sites, the HPC exhibited great adsorption performance of TC in both the batch mode adsorption and the column adsorption process [111]. Recently, Zhang et al. (2022) successfully prepared N-doped HPC via simple one-step carbonization and employed it for TCH adsorption. The sodium dodecylbenzene sulfonate was introduced as a soft template, which optimized the pore structure of the carbon materials, increased the nitrogen content, and added surface functional groups, leading to a high TCH adsorption capacity [112].

**Table 4:** Adsorption characteristics of antibiotics by HPC-based materials.

Hierarchical Porous Carbon Material	Carbon Precursor	Modification Method	Antibiotic	Ad Temp (K)	pH	Time (Min)	Ad Capacity Q_m_ (mg g^−1^)	Refs.
Macro-meso-micro hierarchical porous carbon	Wheat straw	KOH + KMnO_4_ activation	Tetracycline	318	7		584.19	[105]
Fe-doped HPC	Eichhornia crassipes debris	HCl activation, Fe + amino acetic acid synergistic treatment	Tetracycline	318	3–11	10	457.85	[107]
N-doped bifunctional HPC	Glucose hydrochar	KHCO_3_ activation, nitrogen doping	Tetracycline	303	4.85		629.76	[45]
Macro-meso-micro hierarchical porous carbon	Sodium lignin sulfonate	KOH activation	Tetracycline	298	3	360	1297.0	[109]
Micro/meso bimodal porous carbon	Soluble phenolic resin		Tetracycline	298	7		701.31	[111]
N-doped HPC	Soft-templated ZIF-8 for	Nitrogen doping	Tetracycline hydrochloride	298	4.5	900	80.92	[112]
Hierarchical micro/mesoporous carbon	Soybean	KOH activation	Chloramphenicol	298	5	40	892.9	[44]
Hierarchical micro/mesoporous carbon	Corncob	KOH activation	Chloramphenicol	318	9	40	662.3	[44]
Oxygen-enriched HPC	Sodium lignosulfonate	K_2_CO_3_ activation	Chloramphenicol	303	4.86	720	534.0	[108]
Macro-meso-micro hierarchical porous carbon	Sodium lignin sulfonate	KOH activation	Chloramphenicol	298	3–11	360	1067.2	[109]
Hierarchical micro/mesoporous carbon	Sodium carboxymethyl cellulose	KOH activation	Chloramphenicol	298	2–6	60	769.95	[110]
Hierarchical micro/mesoporous carbon	High-salted Spirulina residue	KHCO_3_ activation	Sulfathiazole	298	7	240	218.4	[106]

## 4. Photocatalytic Degradation of Antibiotics by Carbon-Based Materials

In addition to adsorption, photocatalysis is a green and efficient technology used to remove antibiotics. Antibiotics can be effectively degraded into a non-toxic small molecular species by a reactive species (e.g.,·OH, O_2_^−^) produced by photocatalysts under sunlight, visible light, or ultraviolet (UV) light. 

**Figure 3:**
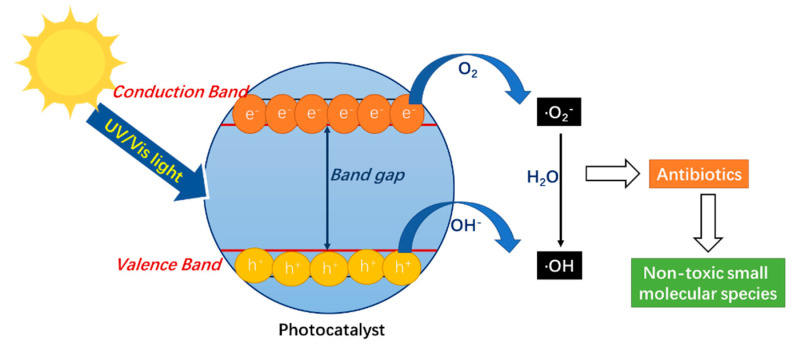
Photodegradation of antibiotics by carbon-based photocatalyst.

The predominant mechanisms of photocatalyst degrading antibiotics can be summarized into three main steps: photon absorption, excitation, and reaction. First, the photocatalyst adsorbs photons equal or superior in energy to its band gap; then, the electrons in the valance band (VB) are activated and jump into the conduction band (CB) where a hole (h^+^_VB_) is produced; finally, the highly reactive positive holes and electrons are separated and migrate to the surface of the photocatalyst, reacting with compounds such as O_2_, OH^−^ and H_2_O groups, and then generating oxidizing species which can degrade antibiotics (Figure 3) [14,113]. Generally, h^+^_VB_ can also directly react with antibiotics, leading to an efficient removal [113]. As for the influencing factors in antibiotic photodegradation, there are three aspects: light source (wavelength), photocatalyst (size, shape, morphology, and dosage), and environment (solution pH, concentration of antibiotics, and coexisting ions and organics) [11].

Semiconducting materials, such as TiO_2_, WO_3_, ZnO, Fe_2_O_3_, CdS, MoS_2_, and graphitic carbon nitride (g-C_3_N_4_), are widely used photocatalysts for water remediation. However, most of them suffer from limitations including large band gaps, instability in an aqueous medium, and high electron-hole recombination rates [114]. To solve these problems, researchers started to combine them with carbonaceous materials to form composites for three main advantages of carbonaceous materials: (1) high surface area, which implies an improved adsorption ability; (2) property of decreasing electron-hole recombination rates, which is because they can effectively scavenge photo excited electrons from the conduction band of semiconducting photocatalysts; (3) as the carrier, co-catalyst, and photosensitizer, abilities to broaden the light absorption range and elevate the photocatalytic activity of the composite [115,116].

### 4.1. Graphene-Based Materials Applied in Photocatalytic Degradation of Antibiotics

Due to its large SSA for uniform dispersion, narrow band-gap energies, high electrical conductivity, and low production cost for mass production, graphene has been thoroughly investigated as a favorable photocatalyst for photodegrading antibiotic pollutants in the aqueous environment. Nevertheless, the self-aggregate process between graphene sheets makes it easy to lose catalytic activity. Therefore, efforts were made to study graphene-based photocatalysts combined with other photocatalysts to achieve a better catalytic performance of antibiotics (Table 5) [53].

Metal oxides are most commonly applied in the photocatalytic degradation of antibiotics. Graphene [117], Go [118], and rGO [119] have all been used to couple with TiO_2_ to increase TiO_2′_s visible light adsorption during photocatalytic degradation. Bi-based composite is another prominent photocatalyst. Bismuth tungsten oxide (Bi_2_WO_6_) [120], bismuth vanadate (BiVO_4_) [121,122], and Br-deficient bismuth oxybromide (Bi_x_O_y_Br_z_) [123,124] are the three most common Bi-based oxides used for construction of hierarchical heterojunction with graphene and its derivatives. Jiang et al. (2022) facilely synthesized (Bi)BiOBr/rGO by employing an in situ reduction strategy, which exhibited >98% degradation efficiency of TC within 20 min. The improved photocatalytic ability was because rGO was able to speed up the electron transfer, further restraining the recombination of the electron-hole pairs and promoting the photodegradation process [123]. Meanwhile, other metal oxide photocatalysts, such as magnetic metal oxides (cobalt ferrite [125], Fe_2_O_3_ [126]), delafossite oxides (AgFeO_2_ [127]) and pyrochlore (La_2_Zr_2_O_7_ [128]), were combined with graphene for their unique properties. For example, cobalt ferrite/rGO porous balls synthesized via modified microfluidic and calcination methods are to be used for efficient OTC degradation (84.7%) under visible light and could be quickly recovered by magnets. This photocatalytic performance was ascribed to a narrow band gap, outstanding light-harvesting properties, low carrier recombination, and high electron-hole separation capacity [125]. Layered double hydroxide was also identified as a promising photocatalyst, and Ce-doped NiAl LDH/rGO composite was fabricated via a one-step hydrothermal method by Gao et al. (2021) and achieved superior degradation of 94% for CIP within 60 min in visible light. The presence of rGO offered larger SSA, suppressed the agglomeration of NiAl_0.85_Ce_0.15_ nanosheets, and accelerated separation of photo-generated charges, leading to an excellent photocatalytic performance [129].

Compared to binary and single photocatalysts, ternary photocatalysts exhibited enhanced photoelectric performance and photocatalytic activity. With a large surface area and excellent conductivity, Graphene has shown great potential as the mediator in ternary Z–scheme heterojunctions. Hence, many combinations of two different semiconductors, whose energy bands could couple with each other, forming effective interfacial charge transfer channels, were studied to be anchored on graphene, with graphitic carbon nitride (g-C_3_N_4_) used most frequently [130,131,132,133,134]. A remarkable work by Wang et al. (2021) described Z-scheme phosphate-doped BiVO_4_/graphene quantum dots/P-doped g-C_3_N_4_ composites with enhanced photocatalytic activity for the degradation of NOR (86.3% within 120 min). The principal reason for such a noteworthy performance was the role of graphene quantum dots as the electron mediator for inhibiting the recombination of photogenerated electron-holes, boosting interfacial charge transfer between phosphate-doped BiVO_4_ and P-doped g-C_3_N_4_, and expanding the range of visible light response [133]. In addition to Z-scheme systems, many other ternary photocatalysts were studied for their advanced properties and facile synthesis. For example, Kumar et al. (2022) prepared ZnO/CdO/rGO nanocomposites by refluxing, which realized 99.28% removal of CIP under UV light for 75 min. The fantastic photocatalytic activity of the material was due to the combination of ZnO and CdO, which expanded the band gap energy, and the presence of rGO, which improved adsorption capacity and generated more reactive oxygen species (ROS) by enhancing electron-hole pair separation [135]. Moreover, BiVO_4_/FeVO_4_@rGO [136] with novel 3D/2D/2D heterojunction structure and bifunctional CeO_2_/CdS/rGO [137] for photodegradation and photoreduction were synthesized by a green and cost-effective one-step hydrothermal method.

**Table 5:** Degradation characteristics of antibiotics by graphene-based photocatalysts.

Photocatalysts	Antibiotic	Dosage (g/L)	Detection Wavelength (nm)	Light Source	Degradation Efficiency	Ref.
(Bi)BiOBr/rGO	Tetracycline	1.0		Visible light	>98% within 20 min	[123]
rGO/Bi_4_O_5_Br_2_	Tetracycline	0.5	356	Visible light	95.2% within 60 min	[124]
α-Fe_2_O_3_/rGO	Tetracycline	5.0		Visible light	99% within 140 min	[126]
La_2_Zr_2_O_7_/rGO	Tetracycline	1.0	357	Visible light	82.1% within 40 min	[128]
Graphene/TiO_2_/g–C_3_N_4_	Tetracycline		357	Visible light	83.5% within 80 min	[130]
g-C_3_N_4_/MnO_2_/GO	Tetracycline	0.5		Visible light	91.4% within 60 min	[131]
15%AgBr/5GO/Bi_2_WO_6_	Tetracycline	0.4	357	Visible light	73.3% within 15 min, up to 84%	[132]
Ag_2_O/Bi_2_WO_6_/rGO	Tetracycline	1.0		Visible light	95.3% within 40 min	[134]
BiVO_4_/FeVO_4_@rGO	Tetracycline	0.6	356	Visible light	91.5% within 100 min	[136]
QDs-BiVO_4_/rGH	Tetracycline hydrochloride	0.5	357	Visible light	73.2% within 120 min	[122]
CF/rGO	Oxytetracycline		354	Visible light	84.7%	[125]
GO/TiO_2_	Amoxicillin	0.6	230	UV light	99.84% within 60 min	[118]
rGO/Bi_2_WO_6_	Norfloxacin	0.5		Visible light	87.49% within 180 min	[120]
BiVO_4_/GQDs/PCN	Norfloxacin	1.0	273	Visible light	86.3% within 120 min	[133]
W-BiVO_4_-x/rGO	Ciprofloxacin	1.0		Visible light	93.6% within 60 min	[121]
NiAlCe LDH/rGO	Ciprofloxacin	0.25	271	Visible light	94% within 180 min	[129]
ZnO/CdO/rGO	Ciprofloxacin	0.5	270	UV light	99.28% within 75 min	[135]
CeO_2_/CdS/rGO	Ciprofloxacin	0.5		Sunlight	90% within 120 min	[137]
AgFeO_2_/GO_3_	Lomefloxacin	0.583	250–450	Visible light	88% within 75 min	[127]

### 4.2. Carbon Nanotube-Based Materials Applied in Photocatalytic Degradation of Antibiotics

CNTs with multiple graphite layers not only have the electron-trapping ability to attract electron pairs that semiconductors emit and increase the efficiency of photocatalytic reactions but also exhibit photosensitization, which can release electrons into the semiconductor’s conduction band, raising the electron-hole density, resulting in efficient charge separation. Thus, CNTs should play essential roles in photocatalytic composites for antibiotics degradation [138]. Table 6 lists recent studies of CNT-based photocatalysts for antibiotics removal.

It was reported that CNTs-based binary photocatalysts have the potential to change nanocrystal structure and superior photocatalytic efficiency. Many photocatalysts, such as TiO_2_ [139], MOFs [140], Bi-based oxides [141,142], and lanthanum vanadate [143], were combined with CNTs for antibiotics photocatalytic degradation. Very recently, Gao et al. (2022) used MWCNTs to induce and optimize the {312}/{004} facet ratio change of Bi_5_O_7_I photocatalysts, and therefore accelerated the transformation of photogenerated charge carriers. As a result, the composites exhibited high photocatalytic oxidation efficiency (88.2%) for representative ofloxacin (OFL) [142]. Another noteworthy work presented by Khazaee et al. (2021) in which CuBi bimetallic alloy nanosheets-supported functionalized MWCNTs were synthesized via a facile one-pot process, and achieved the highest degradation rates of CIP and OFL at only 90 min reaction time. The synergistic effects of the SPR effect of Cu, the boosting of hot electron flux at the Schottky junction caused by the intimate contact between the alloy and nanotubes, and the higher oxidative activity led to a high photocatalytic oxidation rate of the nanocomposite [144]. Moreover, Zuo et al. (2022) constructed mesoporous carbon nanotube networks loaded with trace atomic Fe sites by calcining melamine–cyanuric acid complex and glucose absorbed with Fe^3+^ at high temperatures, which exhibited high photodegradation rates of 93.2%, 99.4% and 94.3% for TCH, CTC and OTC. The single atomic Fe with a Fe-N-C structure improved visible light absorption, and the N-doped CNT networks promoted electron and mass transfer as well as enhanced the fast transfer of degradation products along the inner and outer walls of the tubes [145].

Currently, more researchers are paying attention to the design of ternary photocatalysts based on CNTs, enhancing the removal efficiency of various antibiotics, which mainly are quinolones [138,146], sulfonamides [146], β-lactams [147,148] and tetracyclines [138,149,150]. Zuo et al. (2022) demonstrated the preparation of Z-scheme Ag–AgBr/Bi_2_O_2_CO_3_/CNT heterojunctions, which had an outstanding complete removal of TC within 40 min. CNTs increased visible light absorption, and their electrical conduction promoted the fast transfer of electrons, the increase in carrier density, the decrease in interface transfer resistance, and the corresponding increase in photocurrent, all of which were responsible for the significantly improved photocatalytic performance [138].

**Table 6:** Degradation characteristics of antibiotics by CNT-based photocatalysts.

Photocatalysts	Antibiotic	Dosage (g/L)	Detection Wavelength (nm)	Light Source	Degradation Efficiency	Ref.
Ag–AgBr/Bi_2_O_2_CO_3_/CNT	Tetracycline	0.4		Visible light	100% within 40 min	[138]
MWCNT/TiO_2_	Tetracycline	0.2	360	UV light	100% within 100 min	[139]
HPWx@Fe_2_O_3_-CNTs	Tetracycline	0.25	356	Visible light	100% within 40 min	[149]
Fe-CNTs	Tetracycline hydrochloride	0.05	358	Visible light	93.2% within 100 min	[145]
MWCNT/BiVO_4_	Oxytetracycline	0.25	360	Visible light	88.7% within 60 min	[141]
Fe-CNTs	Oxytetracycline	0.05	355	Visible light	94.3% within 100 min	[145]
Fe-CNTs	Chlortetracycline	0.05	370	Visible light	99.4% within 80 min	[145]
NiFe_2_O_4_/MWCNTs/BiOI	Doxycycline	1.25	351	UV light	92.18% within 300 min	[150]
CNT@MIL-101(Fe)	Ciprofloxacin	0.5		Visible light	90% within 45 min	[140]
CuBi/MWCNTs	Ciprofloxacin			Visible light	93% within 90 min	[144]
MWCNTs-{312}/{004}Bi_5_O_7_I	Ofloxacin	1.0		Visible light	88.2%	[142]
CuBi/MWCNTs	Ofloxacin			Visible light	91% within 90 min	[144]
Bi_2_MoO_6_/Bi_2_WO_6_/MWCNTs	Ofloxacin	2.0		Visible light	91.3% within	[146]
CNTs/LaVO_4_	Sulfamethazine	0.3	255	Visible light	Up to 95% within 90 min	[143]
Bi_2_MoO_6_/Bi_2_WO_6_/MWCNTs	Sulfadimidine	2.0		Visible light	88.8%	[146]
SWCNT/ZnO/Fe_3_O_4_	Cefixime	0.46	280	UV-A light	94.19%	[147]
Bi_2_WO_6_/CNT/TiO_2_	Cephalexin	0.75	262	Sunlight	98.7% within 70 min	[148]

### 4.3. Biochar-Based Materials Applied in Photocatalytic Degradation of Antibiotics

Under light irradiation, biochar can generate various reactive oxygen species (ROS) through biochar carbon matrix (BCM) and dissolved organic matter (DOM) to enhance the indirect photodegradation of pollutants and oxidize various molecules. Yang et al. (2021) revealed that BCs could photodegrade the SMX and CAP through direct and indirect pathways. Specifically speaking, in the low-concentration BC solutions, the oxygen reduction performances of BCs and ·OH radicals generated by DOMs can indirectly photodegrade SMX and CAP by promoting the oxidation process and electron transfer [151]. Furthermore, modification methods were applied to improve the light response performance of BC, such as ball milling was proven to improve the capacities of adsorption and ROS generation by increasing the SSA, pore volume, oxygen-containing functional groups, and the carbon defects of BC [152].

Moreover, BC is a good carrier for photocatalysts as it can bring some advantages: (1) facilitate the photodegradation process due to the high adsorption capacity, (2) expand the light absorption range, and (3) provide an efficient electron-transfer channel and acceptor to enhance the separation of photogenerated electron-hole pairs. So, the combination of BC and semiconductors such as metallic oxides, metallic sulfides, and graphitic carbon nitride were intensively explored for antibiotics removal. For example, (modified) TiO_2_/BC [153,154,155], Bi_2_WO_6_/Fe_3_O_4_/BC [156], PbMoO_4_/BC [157], CdS/BPC [158], and g-C_3_N_4_/BC [159,160] all have high photocatalytic efficiency and long-term use durability because of their high surface area, adequate active sites, and compact interface. Recently, layered double hydroxides/oxides [161,162] and spinel ferrites received great attention as emerging categories of photocatalysts to be combined with BC. Li et al. (2022) prepared ZnO/ZnFe-LDH loaded on BC via a facile hydrothermal method. The BC matrix offered a homogeneous distribution of LDH, narrowed band gap, expanded visible-light adsorption, and enhanced separation of electron-hole pairs, leading to significant photocatalytic degradation of TC (87.7%) [163]. Zinc ferrite (ZnFe_2_O_4_) with narrow band-gap energy of 1.9 eV was combined with B, N co-doped BC by Peng et al. (2021) to generate a large surface area and numerous vacant sites for TCH adsorption and electron capture, and form the tight interface for efficient transfer and mobility of charge carriers [164].

Additionally, it has been shown that a system based on transition metal phosphides is a wise choice for photocatalytic applications. Wang et al. (2022) constructed a photocatalytic system composed of Ni_x_P/biocarbon composite as the reactive center and erythrosine B as a sensitizer for SMX removal. The NixP mixed crystal phases presented excellent photocatalytic activity due to its faster electron transfer ability [165]. Table 7 lists the BC-based photocatalysts mentioned above for antibiotics degradation. 

### 4.4. Hierarchical Porous Carbon-Based Materials Applied in Photocatalytic Degradation of Antibiotics

Carbon materials with a hierarchical architecture are promising photocatalysts as their special interconnected porous structures can enhance the transport of substrates/products and the exposure of active sites so that in subsequent reactions, the charge carrier transfer distance would be shortened, which will promote the separation and migration of photo-excited electron-hole carriers. However, few pieces of research have been conducted on HPC-based photocatalysts for antibiotics removal.

Zhou et al. (2021) reported a novel catalyst of N-doped magnetic three-dimensional hierarchical porous carbon microspheres@TiO_2_ (N-doped MCMs@TiO_2_), which demonstrated impressive photodegradation ability for TC (up to 99.87% within 120 min) under UV irradiation. According to the test results, the catalyst exhibited an extended continuous pore size distribution and a meso/macropore dominant hierarchical structure, which gave TiO_2_ a better supporting matrix and increased the number of catalytic sites. The N-doped carbon structure significantly narrowed the band gap of TiO_2_, increased the adsorption of visible light, and favored the electrons transferring. Above are the reasons for the improved photocatalytic performance of the material [166]. In another study, Wang et al. (2021) prepared metal-organic complex-derived 3D HPC-supported g-C_3_N_4_/TiO_2_ as photocatalysts for chlortetracycline hydrochloride (CTC-HCl) removal. It showed high CTC-HCl photocatalytic efficiency up to 97.8% after 60 min in the static system, which was ascribed to two structural properties: (1) the 3D porous structure with a high SSA could offer multidimensional adsorption-enrichment sites, (2) the heterojunction could promote the separation and migration of the photogenerated electrons and holes [167].

## 5. Conclusions and Outlooks

Along with human activities and industrial development, water pollution issues are becoming increasingly severe, threatening the ecological environment and human health. Due to the overuse of antibiotics in aquaculture and human/animal treatment in recent years, antibiotics are one of the major emerging pollutants in the aquatic environment. From one-dimensional carbon nanotubes, two-dimensional graphene, and three-dimensional biochar to hierarchical porous carbon, carbonaceous materials have been widely used as adsorbents and photocatalysts for eliminating antibiotics. This review discusses recent advances in carbon-based materials for antibiotic adsorption and photodegradation. The main adsorption mechanisms involved in the adsorptive removal process of antibiotics are π-π interactions, electrostatic interaction, and hydrogen bonding. Except for environmental parameters, the adsorption performance of carbon-based materials primarily depends on their pore size, uniformity, and functional groups. Carbonaceous materials have been modified via chemical or physical methods and loaded with functional nanoparticles or compounds to form carbon-based composites for improved adsorption properties. On the other hand, carbon-based materials have been applied in antibiotic photodegradation as photocatalyst carriers, co-catalysts, and photosensitizers. The predominant steps for antibiotic photocatalytic degradation are photon absorption, excitation, and reaction, depending on three main species, O_2_^−^, h^+^, and OH radicals. The carbon-based photocatalysts usually show better adsorption performance, a higher ability to scavenge photo-excited electrons, and broadened light absorption range.

Even though many studies were conducted on the adsorptive and photocatalytic removal of antibiotics by carbon-based materials, many research gaps and uncertainties remain. The main challenges to overcome in future research are as follows.

1. Hastening the formation of antibiotic-resistance genes and antibiotic-resistant bacteria is the main pathway for antibiotics to harm the ecosystem. However, using carbon-based materials to disrupt antibiotic resistant genes and alter bacterial resistance has been rarely reported. Further research should be conducted to determine the impact of carbon-based materials on antibiotic resistance.
2. Some carbon-based materials have intrinsic toxicity, and others can produce toxic by-products when removing antibiotics, such as the leaching of metal ions. So, they may have a negative impact on the water environment. The long-term fate and environmental risks of carbon-based materials in the aqueous environment are still unclear, and further studies of their toxicity and biological responses are needed.
3. Despite intense research, antibiotics’ adsorption and photodegradation mechanisms remain vaguely interpreted, as conclusions based on partial characterization analysis and traditional models are not accurate or comprehensive. Issues like the synergistic effect of the components in the carbon-based nanocomposites, the effect of the antibiotic species on the removal properties, and the applicability of the relevant models need to be further investigated.
4. Currently, most studies on the removal of antibiotics by carbon-based materials are limited to batch experiments at the laboratory scale rather than the pilot scale, resulting in a gap between research and application. The experiments were usually conducted in mixed antibiotics or antibiotics-metals systems instead of a complex system with coexisted multi-pollutants. From an application point of view, it is vital to test the effectiveness of carbon-based materials for antibiotic removal in a system that resembles a natural water environment, and to investigate other pollutants’ influence on the removal process. More attention should be paid to interactive mechanisms among antibiotics, interfering substances, and carbon-based materials. Furthermore, for large-scale engineering applications, in addition to the removal properties of the materials, their mass production feasibility and economic efficiency should be considered, such as raw materials, production cost, production cycle, and yield.

## Figures and Tables

**Table 7:** Degradation characteristics of antibiotics by BC-based photocatalysts.

Biomass	Pyrolysis Temp (°C)	Photocatalysts	Antibiotic	Dosage (g/L)	Light Source	Degradation Efficiency	Refs.
Poplar sawdust	500	PbMoO_4_/BC	Tetracycline	3.0	Visible light	61.0% within 120 min	[157]
Potato stems and leaves	600	CdS/BPC	Tetracycline	0.1	Visible light	84.6% within 120 min	[158]
Enteromorpha	700	g-C_3_N_4_/BC	Tetracycline	0.2	Visible light	88% within 60 min	[159]
Caragana korshinskii	650	K-g-C_3_N_4_/BC	Tetracycline	1.0	Visible light	90.94% within 180 min	[160]
Rice husks	400	ZnO/ZnFe-LDH/BC	Tetracycline	0.2	Visible light	87.7% within 240 min	[163]
Sugarcane bagasse	600	ZnFe/BN-BC	Tetracycline hydrochloride	0.33	Sunlight	98.19% within 120 min	[164]
Rice straw	700	Pure BC	Sulfamethoxazole	0.5	Visible light	96.28% within 720 min	[151]
Reed straw	500	Zn-TiO_2_/pBC	Sulfamethoxazole	1.25	Visible light	81.21% within 180 min	[153]
Baker’s yeast	900	Ni_x_P/BC	Sulfamethoxazole	0.4	Visible light	98.71% within 120 min	[165]
Primary paper mill sludge	800	Mag-TiO_2_/KBC	Sulfadiazine	0.1	Sunlight	t_1/2_= 5.6 ± 0.4 h	[155]
Rice straw	700	Pure BC	Chloramphenicol	0.5	Visible light	95.23% within 720 min	[151]
Caragana korshinskii	650	K-g-C_3_N_4_/BC	Chloramphenicol	1.0	Visible light	82.42% within 180 min	[160]
Poplar woodchips	300	Ball-milled BC	Enrofloxacin	0.2	Visible light	Up to 82.5% within 150 min	[152]
Corn stalk	500	TiO_2_/KBC	Enrofloxacin	2.5	UV light	85.25% within 60 min	[154]
Reed straw	500	Bi_2_WO_6_/Fe_3_O_4_/BC	Ofloxacin	0.4	Visible light	83.1% within 60 min	[156]
Reed straw	500	Bi_2_WO_6_/Fe_3_O_4_/BC	Ciprofloxacin	0.4	Visible light	91.5% within 60 min	[156]
Caragana korshinskii	650	K-g-C_3_N_4_/BC	Norfloxacin	1.0	Visible light	83.62% within 180 min	[160]
husks and paper sludge	500	Zn-Co-LDH/BC	Gemifloxacin	0.75	UV light	92.7% within 100 min	[161]
Palm seeds	600	MnFe-LDO/BC	Metronidazole	0.5	UV light	98% within 60 min	[162]

## Data Availability

Not applicable.
